# Extremely Large Fluid Collection in the Superior Aortic Recess Misdiagnosed as Mediastinal Tumors: A Report of Two Cases

**DOI:** 10.7759/cureus.66331

**Published:** 2024-08-06

**Authors:** Sachie Koike, Takayuki Shiina, Keiichirou Takasuna, Akane Kato, Koudai Komatsu

**Affiliations:** 1 Thoracic Surgery, Ina Central Hospital, Ina, JPN; 2 Respiratory Medicine, Ina Central Hospital, Ina, JPN

**Keywords:** cephalad extension, cystic tumor, mediastinal, fluid collection, superior aortic recess

## Abstract

The superior aortic recess is one of the superior portions of the transverse sinus which is located around the ascending aorta. The fluid collection of the superior aortic recess is sometimes revealed on chest computed tomography, and it becomes more difficult to differentiate from a cystic tumor or lymphadenopathy when the amount of collected fluid is large or the fluid is extended into another area. We report two cases of fluid collection in the superior aortic recess which was misdiagnosed as a cystic mediastinal tumor that underwent surgical resection. An extremely large amount of fluid collection and cephalad extension led us to this clinical course.

## Introduction

The pericardium is a sac that envelopes the heart, except at the two orifices, corresponding to the entrances and exits of the arterial trunks and veins surrounded by their pericardial reflections [1]. The pericardial reflections form small pockets, in which fluid can collect. These pockets are called pericardial sinuses or recesses, which include the transverse sinus, oblique sinus, postcaval recess, and pulmonary venous recess [2]. The embryological development of the transverse sinus has been reported as follows: 1) the heart tube is anchored to the body wall by both arterial and venous poles and dorsal mesocardium; 2) the pericardium anterior to the heart tube developed to the dorsal part with the dissolution of the dorsal mesocardium; 3) the pericardium surrounds the heart tube, and the dorsal part between the arterial pole and the venous pole becomes transverse sinus [1]. It develops the cephalad with the development of the arterial pole and it reaches the level of sternal angle [3,4]. The transverse sinus can be divided into four parts as follows: superior aortic recess, inferior aortic recess, and left and right pulmonic recesses [2]. The superior aortic recess is the most superior division of the transverse sinus and it extends upward along the right side of the ascending aorta, usually to the level of the sternal angle [4]. The fluid collection in the superior aortic recess showed varying appearances, ranging from bands to mass-like densities that could be misinterpreted as mediastinal lymph node enlargements, cystic mediastinal masses, and aortic dissections on computed tomography (CT) scans [3]. Herein, we report the case of two patients whose chest CT showed extremely large amounts of fluid collection in the superior aortic recess, which was misdiagnosed as cystic mediastinal mass and underwent surgical resection.

## Case presentation

Case 1

**Figure 1:**
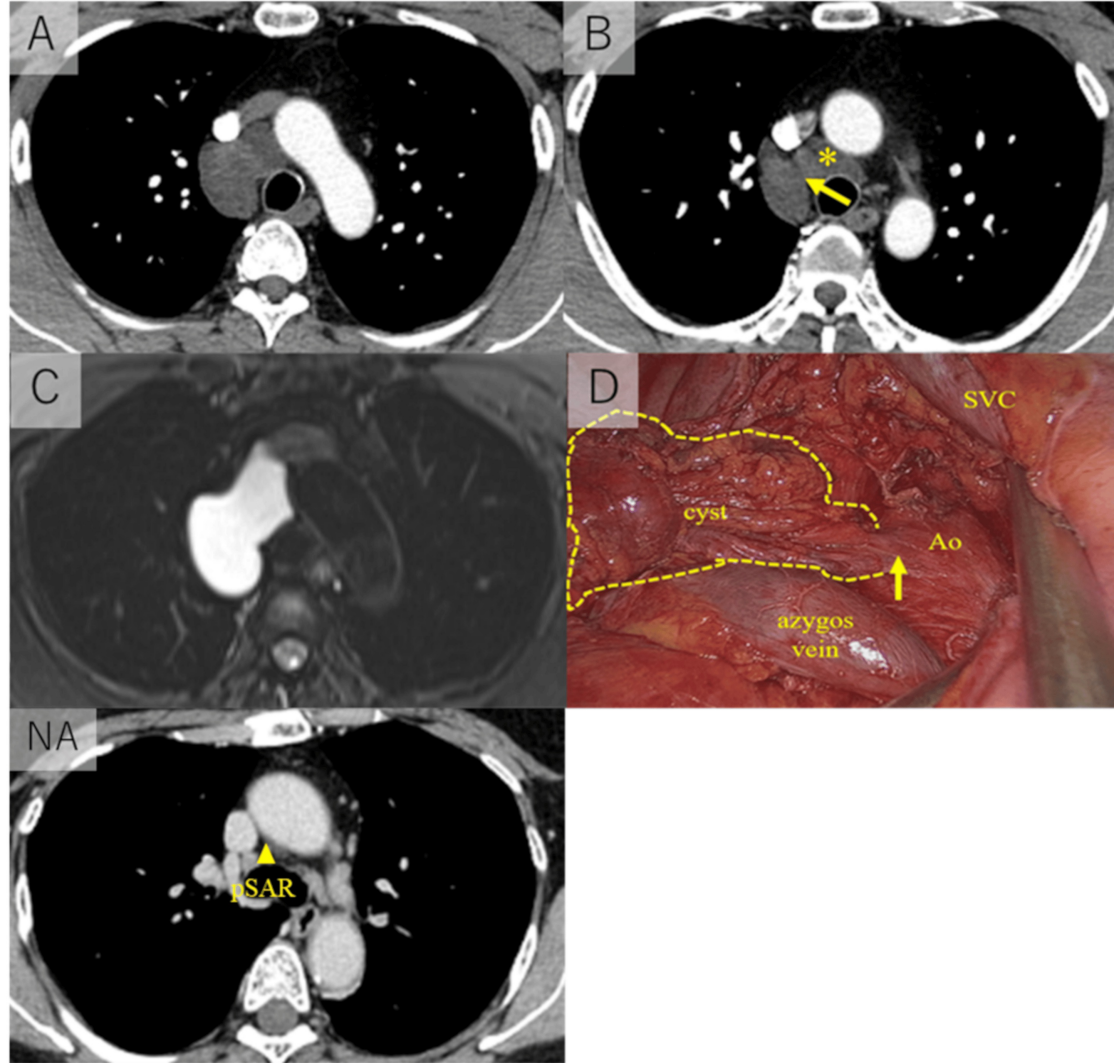
Radiological images and operative findings of Case 1 A: The fluid density mass (5.8 x 3.5 cm) is located in the right paratracheal space of superior mediastinum. B: The origin of the lesion was the fluid collection of the posterior portion of the superior aortic recess (*). The fluid collection extended the cephalad to the right paratracheal space of the superior mediastinum through the yellow arrow route. C: Magnetic resonance image (MRI) of the mass showed a high density on T2-weighted imaging. D: The cystic tumor is connected to the space behind the aorta (yellow arrow = connected part). NA: The normal anatomy figure (reference). The yellow arrowhead is in the pSAR. SVC = superior vena cava; Ao = aorta; pSAR = posterior portion of the superior aortic recess.

**Figure 2:**
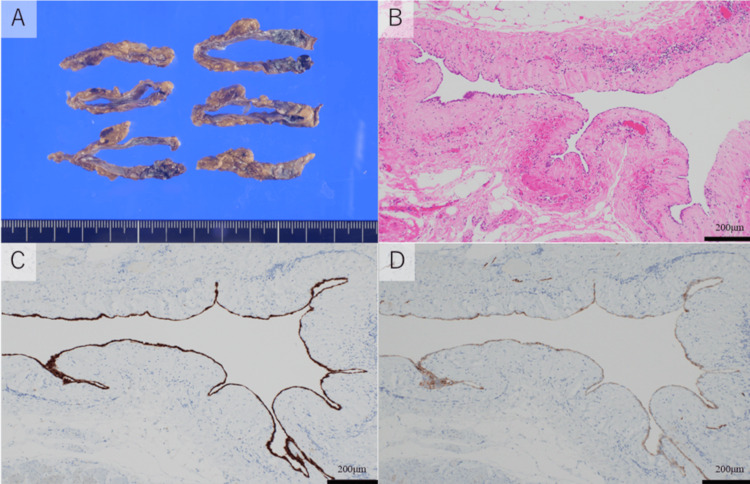
Pathological findings of Case 1 A: Cystic lesion. B: The wall of the lesion consisted of simple lining cells surrounded by fibrous tissue (hematoxylin and eosin ×100). C: The lining cells inside the cyst were positive for calretinin. D: Podoplanin (D2-40) (immunostaining ×100).

A 43-year-old man with no previous medical or surgical history was referred to our hospital due to an abnormality on a chest X-ray during a medical checkup. CT revealed a fluid density mass (5.8 x 3.5 cm) located in the right paratracheal space of the superior mediastinum (Figure 1A). No cardiomegaly or pulmonary edema was found. Magnetic resonance image (MRI) of the mass showed a high density on T2-weighted imaging (Figure 1C) and a low density on T1-weighted imaging, which suggests a cystic lesion. We suspected the lesion was a cystic tumor such as a bronchogenic cyst and decided to schedule video-assisted thoracoscopic surgery (VATS) for the treatment and diagnosis. During the surgery, we found the cystic tumor connected to the space behind the aorta (Figure 1D). We resected the cyst without the part that was connected to the space behind the aorta. Pathological examination found that the immunostaining revealed that the lining cell inside the cyst was positive for calretinin and podoplanin (D2-40) (Figure 2A-2D). These results suggested that the cells were mesothelial cells and the cystic tumor was a part of the pericardium. We rechecked the preoperative images and intraoperative findings and finally diagnosed the lesion as a fluid collection of the posterior portion of the superior aortic recess, which extended cephalad to the right paratracheal space of the superior mediastinum (Figure 1A, 1B).

Case 2

**Figure 3:**
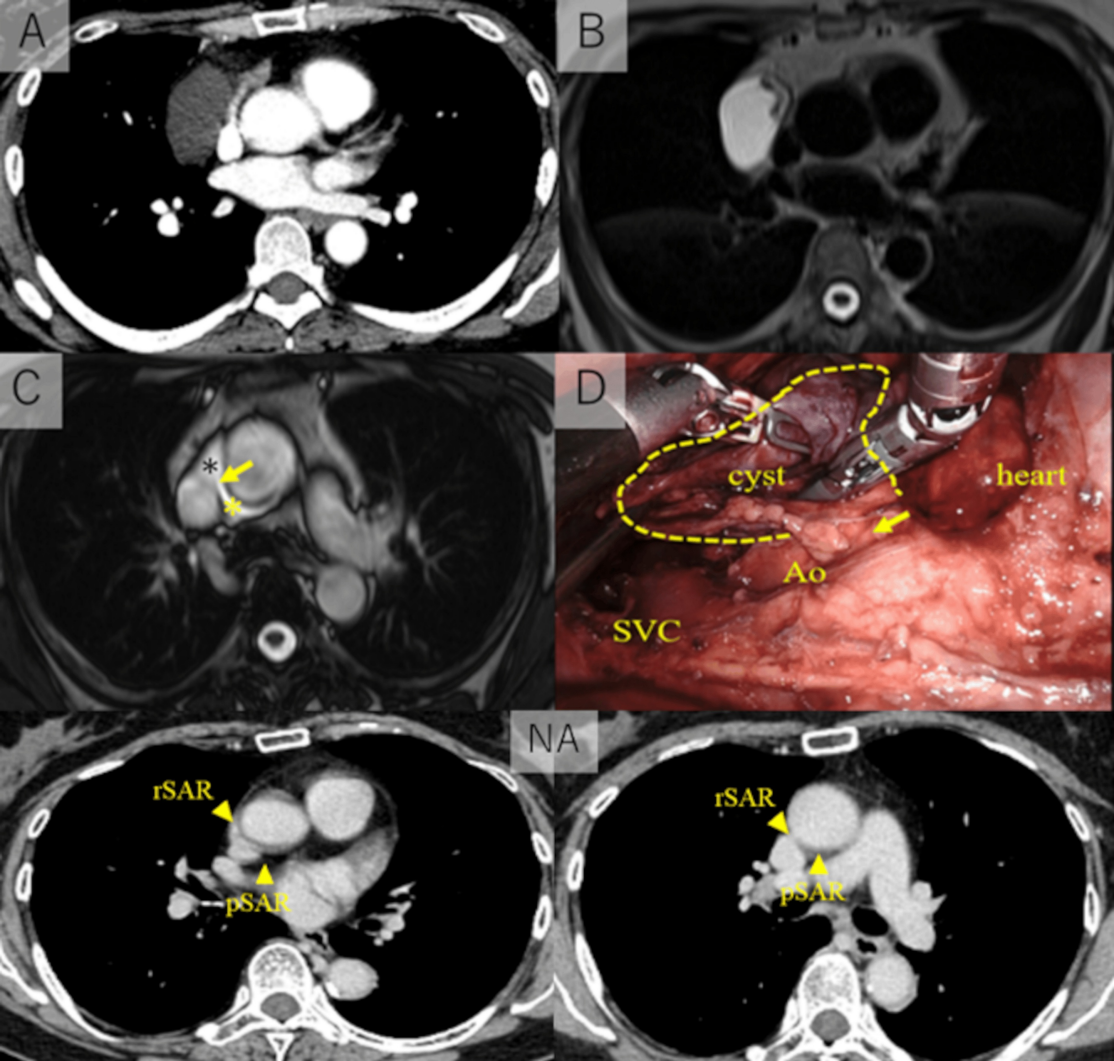
Radiological images and the operative findings of Case 2 A: Computed tomography revealed a fluid density mass (5.1 x 2.9 cm) in thymus. B: Magnetic resonance image (MRI) of the mass showed a high density on T2-weighted imaging. C: The origin of the lesion was a fluid collection of the right lateral portion of the superior aortic recess (black *). The fluid collection is connected to the fluid collection of the posterior portion of the superior aortic recess (yellow *) through the yellow arrow route. D: The cystic tumor is connected to the space in front of the aorta (yellow arrow = connected part). NA: The normal anatomy figure (reference). Yellow arrowheads are in the pSAR and the rSAR. SVC = superior vena cava; Ao = aorta; pSAR = posterior portion of the superior aortic recess; rSAR = right lateral portion of the superior aortic recess.

**Figure 4:**
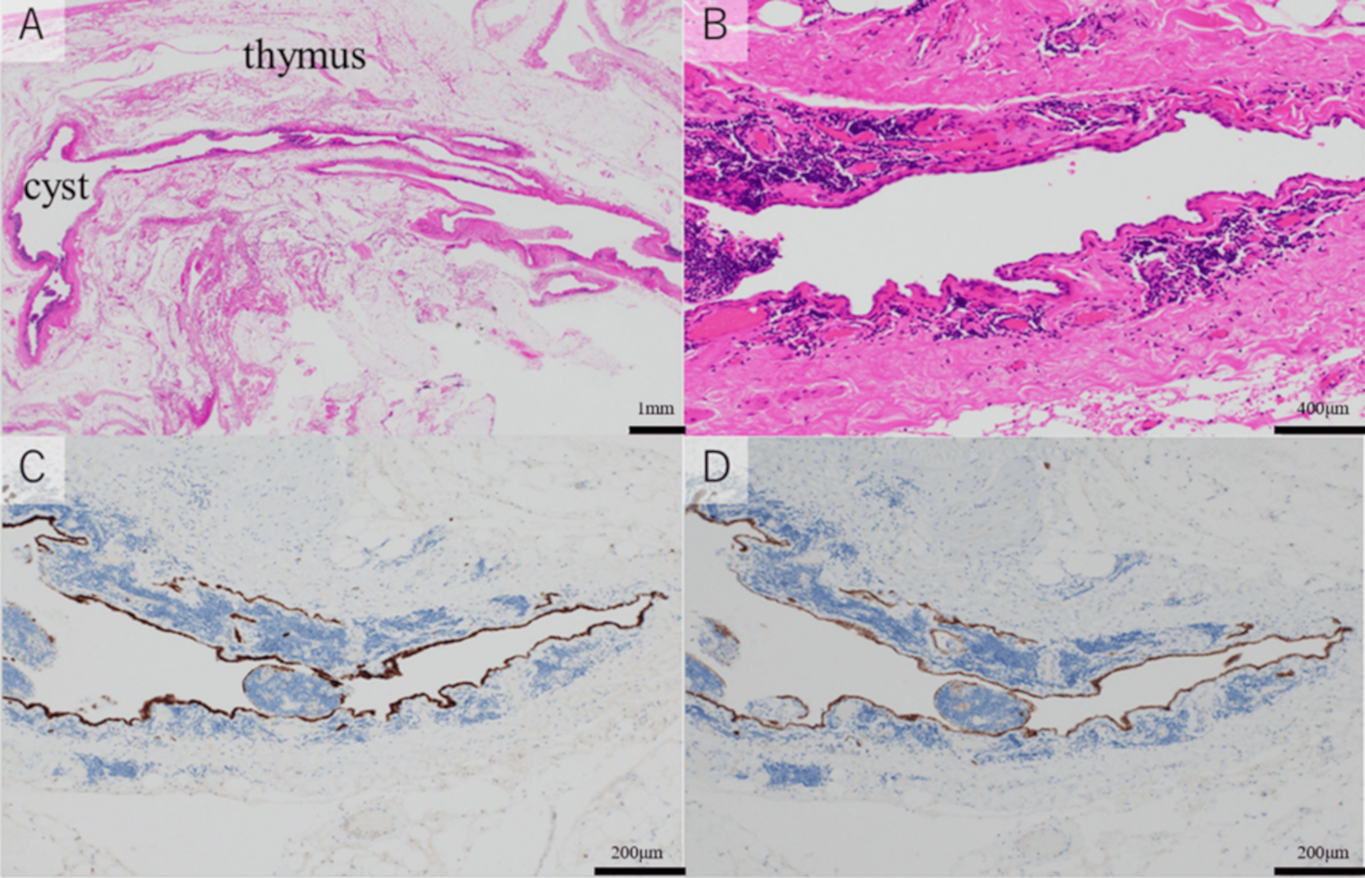
Pathological findings of Case 2 A: The cystic lesion was in the thymus. B: The wall of the lesion consisted of simple lining cells surrounded by fibrous tissue (hematoxylin and eosin ×100). C: The lining cells inside the cyst were positive for calretinin and D: podoplanin (D2-40) (immunostaining ×100).

A 51-year-old woman with no previous medical or surgical history was referred to our hospital due to an abnormality on her chest X-ray during a medical checkup. CT revealed a fluid density mass (5.1 × 2.9 cm) in the thymus (Figure 3A). No cardiomegaly or pulmonary edema was found. MRI of the mass showed a high density on T2-weighted imaging (Figure 3B) and a low density on T1-weighted imaging, which suggested a cystic lesion. We suspected that the lesion was a thymic cyst and scheduled a robotic thymectomy. During the surgery, we found that the cystic tumor originated from the pericardium in front of the aorta, and connected to the aorta (Figure 3D). We resected the cyst and sutured the defective part of the pericardium. Pathological examination found that the immunostaining showed that the lining cells within the cyst were positive for calretinin and podoplanin (D2-40) (Figure 4A-4D). These results suggested that the cells were mesothelial cells and the cystic tumor was a part of the pericardium. We rechecked the preoperative MRI image and found that the cystic lesion contained a large amount of fluid collection in the right lateral portion of the superior aortic recess, and it connected to the fluid collection of the posterior portion of the superior aortic recess (Figure 3B, 3C).

## Discussion

The superior aortic recess is one of the pericardial recesses located in the most superior division of the transverse sinus of the pericardium [4]. It can be divided into three portions: the right lateral portion, anterior portion, and posterior portion [3,4]. Fluid collection in the superior aortic recess is sometimes revealed on chest CT, and the appearance simulates aortic dissection, mediastinal mass, or lymphadenopathy [2-4]. Knowledge of these appearances can prevent misinterpretation of a normal pericardial recess as an abnormality. However, cephalad extension or a large amount of fluid collection makes it difficult to differentiate between the normal pericardial recess and a cystic tumor or lymphadenopathy [3,4]. Choi et al. reported that the fluid collection in the posterior portion of the superior aortic recess usually manifests as a half-moon-shaped fluid collection adjacent to the posterior wall of the ascending aorta which sometimes can extend the cephalad into the right paratracheal region. This extension is likely to occur when the short-axis diameter of the recess is more than 10 mm, and the extension makes it more difficult to distinguish between the recess from a cystic tumor or lymphadenopathy [4]. Also, Winer-Muram et al. reported a case of a 10 mm fluid collection in the superior aortic recess which mimicked a mediastinal mass [5].

In the present cases, the diameter of the fluid collection in Case 1 was 5.8 x 3.5 cm and Case 2 was 5.1 x 2.9 cm. Case 1 was the largest size of fluid collection in the posterior portion of the superior aortic recess among the reported cases [3,4], and Case 2 was one of the largest fluid collections in the right lateral portion of the superior aortic recess (12-56 mm was reported by Yoo et al.) [6]. These extremely large amounts of fluid collection in the present cases led us to misinterpret the images as cystic tumors. In addition, in Case 1, the cephalad extension of the fluid collection into the right paratracheal region, which mimicked a bronchogenic cyst or lymphadenopathy of the superior mediastinum, exacerbated the misdiagnosis.

CT and MRI images of the fluid collection in the superior aortic recess, which facilitated the differentiation from cystic tumors, were reported as follows: 1) slit-like fluid connection between the right lateral portion and the posterior portion; 2) connection between the cephalad extended fluid collection and the posterior portion of the superior aortic recess; 3) without definable walls [3,4]. We retrospectively found 1) the image on MRI of Case 2 and 2) the image on CT of Case 1.

## Conclusions

In conclusion, we have encountered two cases of fluid collection in the superior aortic recess which were misdiagnosed as cystic mediastinal tumors which underwent surgery. An extremely large amount of fluid collection and cephalad extension led us to this misinterpretation. Careful checking of CT or MRI images and obtaining knowledge of images that facilitate distinguishing between a fluid collection of pericardial recess from abnormalities is important to avoid misdiagnosis.
